# Electromyographic investigation of anterior and posterior regions of supraspinatus: a novel approach based on anatomical insights

**DOI:** 10.1080/23335432.2017.1364667

**Published:** 2017-12-15

**Authors:** Soo Y. Kim, Jong Bum Ko, Clark R. Dickerson, David F. Collins

**Affiliations:** a School of Physical Therapy, College of Medicine, University of Saskatchewan, Saskatoon, Canada; b College of Kinesiology, University of Saskatchewan, Saskatoon, Canada; c Faculty of Applied Health Sciences, Department of Kinesiology, University of Waterloo, Waterloo, Canada; d Faculty of Physical Education and Recreation, Neuroscience and Mental Health Institute, University of Alberta, Edmonton, Canada

**Keywords:** Rotator cuff, shoulder, glenohumeral, indwelling, muscle recruitment, partitioning

## Abstract

Supraspinatus is composed of anterior and posterior regions that are distinct. To date, the relative electromyographic (EMG) activity of these regions during different tasks has not been investigated. This work, thus, evaluated activity of the anterior and posterior regions of supraspinatus during isometric actions in different postures. Data were analyzed from 11 healthy participants. Fine-wire electrodes were inserted into the anterior and posterior regions of supraspinatus. EMG activity was recorded during isometric abduction and external rotation exertions against 5% of body weight resistance. Three postures for abduction (30°, 60°, and 90° of humeral abduction, scapular plane) and two for external rotation (0° and 90° humeral abduction) were tested. Each participant’s data were normalized to the peak root mean square (RMS) values for the corresponding region. The RMS of the anterior region was divided by that of the posterior to calculate muscle activation ratios. Non-parametric statistics were used for analyses. The median ratio was lower during external rotation at 90° abduction compared to abduction at 30° (*P* = 0.003). These results suggest that the two regions of supraspinatus are functionally distinct during isometric tasks. The posterior region may play a more dominant role in postures with higher degrees of abduction and during external rotation exertions.

## Introduction

Over the past two decades, our understanding of the supraspinatus muscle has evolved. Supraspinatus is a complex muscle consisting of two anatomically distinct regions: anterior and posterior, based on the lateral attachment of muscle fiber bundles onto the tendon (Vahlensieck et al. 1994; Roh et al. 2000; Ward et al. 2006; Kim et al. 2007). The fiber bundles of the anterior region are pennated and account for 75–86% of the muscle volume (Roh et al. 2000; Kim et al. 2007). Its fibers attach laterally to the thick and narrow part of the anterior supraspinatus tendon (Roh et al. 2000; Kim et al. 2007). The smaller posterior region has a more parallel fiber bundle orientation and attaches laterally to the broader posterior supraspinatus tendon (Roh et al. 2000; Kim et al. 2007).

Targeted studies have investigated functional and morphological aspects of these regions of supraspinatus which challenge the existing notion that the muscle functions as a single collective unit without partitions that diverge functionally. In a real-time ultrasound study, Kim, Bleakney et al. (2010) found significant differences in fiber bundle length changes between the anterior and posterior regions during active shoulder movements. Later, the posterior region was shown to have a higher percentage of Type II (fast) fibers (Kim et al. 2013). Most recently, in a three-dimensional computer modeling study of the innervation pattern of the suprascapular nerve, Hermenogildo et al. (2014) reported that anterior and posterior regions are innervated by distinct primary nerve branches. Such distinct innervation enables each region to be modulated independently both in the magnitude and the direction of force applied to the tendon (English et al. 1993). Clinical implications of these anatomic and histologic findings are widespread as it can impact how rehabilitation specialists analyze and treat movement abnormalities. Rather than investigating the action of the entire muscle with electromyography (EMG), selective differences and synergistic activities of anatomically defined partitions can be investigated. Similarly, treating each of these partitions separately during rehabilitation with modalities such as functional neuromuscular electrical stimulation may have potential for improved clinical outcomes for patients following shoulder surgery or with shoulder subluxation following a stroke.

The study of muscle function through fine-wire EMG is an established method for quantifying muscle recruitment. Although many EMG studies of the supraspinatus exist, only the anterior region has been investigated according to the reported electrode placements. EMG has not been recorded from the posterior region, precluding interpretation regarding its role in arm function. Commonly referenced resources for electrode placement suggest the fine-wire electrode be placed at the medial one-third of the muscle belly, immediately superior to the scapular spine – which represents the anterior region of the muscle belly (Perotto 1975; Basmajian 1978). Importantly, these references were published prior to recognition that the supraspinatus muscle is actually a composite of two smaller sub-volumes that may be functionally distinct.

Although activity of the posterior region has not been investigated using EMG, its function can be postulated based on its morphology. For example, despite the relatively smaller volume of the posterior region compared to the anterior, its fiber bundles attach to a broader portion of the supraspinatus tendon compared to the anterior region. The posterior aspect of the supraspinatus tendon accounts for 53% of the total tendon cross sectional area (Gates et al. 2010). The mean width of the posterior tendon is twice that of the anterior tendon, being approximately 1.6 and 0.8 cm, respectively (Kim et al. 2007). Given its broader tendinous attachment, it has been theorized the posterior region likely plays an important role on the rotator cuff complex. Based on the parallel fiber bundle arrangement and fiber-type composition, the posterior region may be involved in quickly adjusting tension on the rotator cuff during dynamic movement (Kim et al. 2007, 2013). Quick adjustments of the tendon may be particularly important in elevated arm postures and with overhead activities where any buckling of the tendon due to the muscle’s shortened position could be at risk of impingement in the subacromial space.

Additionally, the posterior tendon of supraspinatus shares its lateral attachment onto the middle facet of the greater tuberosity with the infraspinatus tendon. This overlapping is absent for the anterior tendon of supraspinatus (Minagawa et al. 1998). Thus, the posterior region may be more active with external rotation tasks compared to the anterior and perhaps coordinates with infraspinatus.

Patterns of degeneration and morphological changes of the posterior region that occur after injury support the above proposed functions of the posterior region. For example, degenerative rotator cuff tears most commonly involved the posterior region (Kim, Dahiya et al. 2010) and the entire posterior region was absent in cadaveric specimens with extensive rotator cuff tears (Kim et al. 2015). If one of the main roles of the posterior region is to prevent buckling of the tendon in elevated arm postures, then dysfunction or altered activity of the region may be related to tendon pathology patterns. Thus, developing an EMG protocol to investigate this neuromuscular compartmentalization may help elucidate both normal and pathologic muscle function of each region. This would provide insights for clinicians to re-evaluate current assessment and treatment methods for supraspinatus tendon pathology and may clarify the pathophysiology of rotator cuff injuries.

The purpose of this study was to investigate the relative EMG activity of the anterior and posterior regions of supraspinatus during isometric actions for several shoulder abduction and external rotation postures. Based on their distinct morphology and innervation, we hypothesized that the relative activity of the regions would differ significantly between the different postures. Accordingly, we hypothesized the relative activity of the posterior region would be greater than the anterior region during tasks involving greater degrees of active shoulder abduction and during external rotation tasks compared to abduction.

## Methodology

Twelve participants from the University of Saskatchewan campus were recruited for this study (5 M/7F). The mean age of participants was 32.5 ± 12.2 years with a range of 22-61. All participants completed a standardized health screening questionnaire to identify the following exclusion criteria: history of cervical spine or shoulder injury/pathology; degenerative/neuromuscular conditions; hemorrhagic disorders; cardiovascular conditions. Participants taking medications that increased the risk of hemorrhage with needle insertion were also excluded. Demographic details (age and gender), body mass (kg) and hand dominance as determined by the Waterloo Handedness Questionnaire of eligible participants were recorded. All procedures were performed using the arm of the dominant hand with participants seated in a chair without arm rests, back supported and feet flat on the floor. The University of Saskatchewan Biomedical Research Ethics Board approved this study (Bio# 13-44). Written informed consent was obtained from all participants prior to data collection.

Two pairs of fine-wire electrodes were inserted into the supraspinatus, one pair in the anterior region and the other in the posterior. For the first six participants, each electrode was inserted separately using a 2.5″ 25 gauge hypodermic needle (Quinke Point, Kimberly-Clark Spinal QP Needle). For the last six participants, to reduce the number of needle insertions, two electrodes were twisted together with the ends bent at different lengths (3 mm and 5 mm) and were inserted into the muscle using a single 3.5″ 23 gauge hypodermic needle (Quinke Point, Kimberly-Clark Spinal QP Needle). Each electrode was made from a Teflon-coated stainless wire (50.8 μm, coated 114.3 μm; A-M Systems, Sequim, WA, U.S.A; Product 790600) that had 2–3 mm of insulation stripped from the distal end of each wire to record ensemble EMG activity from each portion of the muscle and not single motor unit activity. The inter-electrode distance was approximately 2 mm. All electrodes were gas sterilized at Central Sterilization Services at the University of Alberta Hospital.

**Figure 1.**
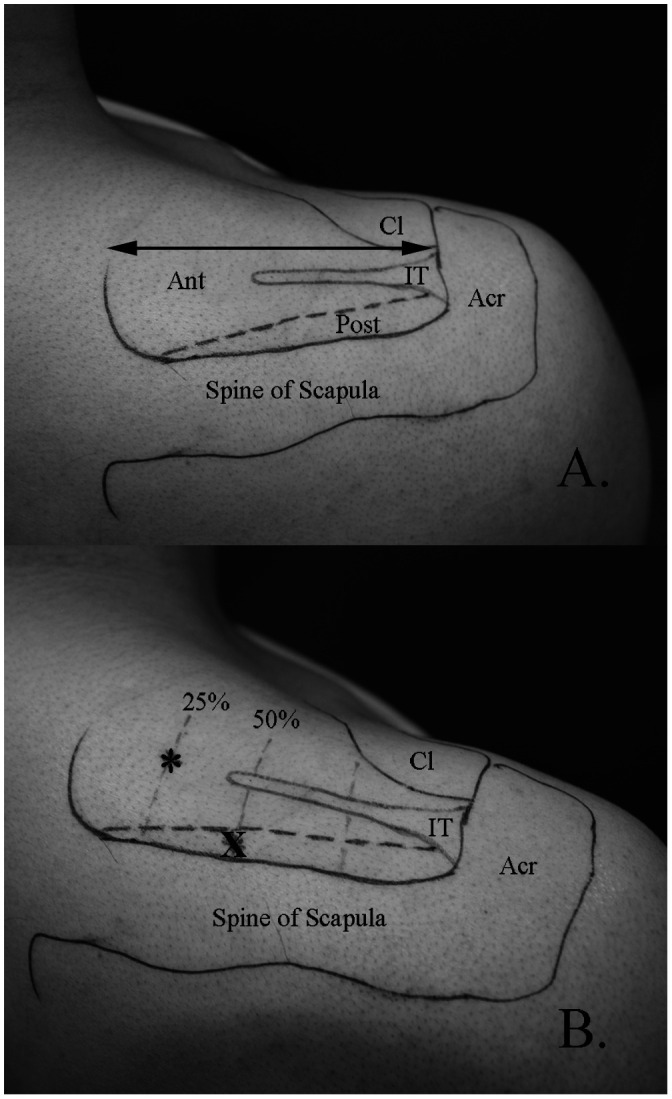
A. External and internal landmarks for electrode placement.

Notes: Cl: clavicle; Acr: acromion; IT: intramuscular tendon — approximate division of anterior and posterior regions of supraspinatus; ↔ muscle length. B. Electrode placement for anterior and posterior regions. * electrode placement for anterior region; X electrode placement for posterior region; 25% of muscle length from the medial aspect; 50% of muscle length.

To guide intramuscular electrode placement, external and internal landmarks and reported details of the anatomic divisions between the anterior and posterior regions along with their respective parts; superficial, middle, and deep, were used (Kim et al. 2007). External landmarks included the acromion, spine of scapula, and clavicle. These landmarks were palpated and then outlined on the skin of each participant using a marker. To determine the approximate anatomic division of the anterior and posterior regions of supraspinatus, internal landmarks including the intramuscular tendon and the supraspinous fossa were identified with real-time ultrasound imaging (12 MHz linear array transducer, GE Logic E, GE Medical Systems, Milwaukee, Wisconsin, U.S.A) by the principal investigator (S.Y.K). The approximate length and position of the intramuscular tendon was marked on the skin (Figure 1(A)). Next, the length of the muscle, defined as the distance from the medial aspect of the acromion to the most medial aspect of the muscle belly, was measured.

**Figure 2.**
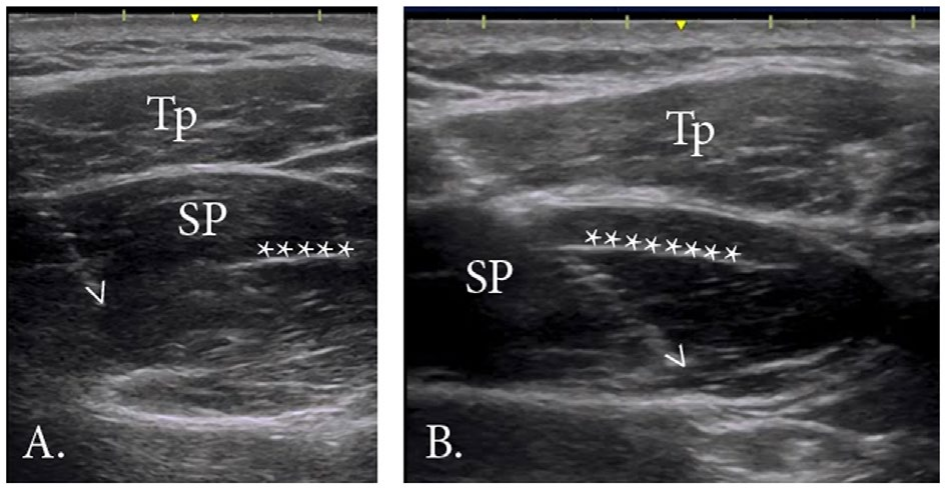
Fine-wire electrode placement.

Notes: Ultrasound guided insertion into the anterior region (A) and posterior region (B). Tp = trapezius; SP = supraspinatus; ***** intramuscular tendon; tip of needle.

All electrodes were inserted under the guidance of real-time ultrasound imaging, which has been shown to be an accurate and repeatable method of intramuscular electrode placement (Hodges et al. 1997). The primary author (S.Y.K) performed all insertions. Prior to insertion, the area was cleaned with 90% rubbing alcohol and allowed to dry. See Figure 1(B) for electrode insertion points. For the anterior region, the needle was inserted at point * (25% of the muscle length from the medial aspect). The needle was advanced from a medial to lateral direction, angled at approximately 30 degrees in the coronal plane (Figure 2(A)). The tip of the needle was advanced until it reached approximately 50% of the muscle depth. For the posterior region, the needle was inserted at point X (~0.5 cm anterior to the spine of the scapula, 50% of the muscle length). The needle was angled at approximately 30 degrees in the coronal plane, following the line of the scapular spine and advanced until the tip reached approximately 50% of the region’s depth (Figure 2(B)).

After insertion, the wires extended approximately 8 cm beyond the surface of the skin and were secured with Transpore™ Medical Tape near the insertion site to minimize movement artifacts. Floating spring adapters (Noraxon, Scottsdale, AZ, U.S.A) were attached to each electrode and then connected to snap EMG leads (Noraxon, Scottsdale, AZ, U.S.A). The leads were secured using doubled-sided tape. Each participant was asked to perform several repetitions of moderate-level isometric and dynamic contractions after the insertion of wires to ensure they were securely embedded in the supraspinatus and that participants could contract the muscle without discomfort.

To later normalize data, EMG signals were recorded from the anterior and posterior regions of supraspinatus during maximum voluntary isometric contractions (MVIC). Participants performed two sets of MVICs of the test arm in six different positions: 0°, 30°, 60°, and 90° of humeral abduction in the scapular plane (elbow extended), and humeral external rotation in 0° and 90° glenohumeral abduction (elbow flexed to 90°) . The testing order was randomized. Each exertion was held for 3 s against a portable hand-held dynamometer (Lafayette Manual Muscle Tester, Model 01163, IN, USA), placed proximal to the radial styloid process. To prevent fatigue, a 2 min rest was provided between each exertion (Mathiassen et al. 1995).

For the testing protocol, EMG activity from the anterior and posterior regions of supraspinatus was recorded during isometric abduction and external rotation exertions in different postures. For the abduction exertions, we tested three postures: 30°, 60°, and 90° of humeral abduction in the plane of the scapula. For the external rotation exertions, we tested two postures: 0° and 90° of humeral abduction. Each participant was asked to hold the isometric exertion contraction against a resistance of 5% of his/her body weight (3.6 ± 1.5 kg, range 2.7–5.1 kg) provided by a hand held weight or Thera-band®. Thera-band® was used to provide resistance for the external rotation exertion at 0° abduction; the hand held weight was used for all other exertions. To determine the appropriate Thera-band® color and percent elongation of the band to provide the 5% body weight resistance, the guidelines provided by Page et al. (2000) were used. One end of the Thera-band was wrapped around the participant’s hand and the other end was held by the research assistant. The participant was asked to hold their arm at 0° abduction with their elbow bent to 90° while resisting the internal rotation force applied through the Thera-band® by the research assistant. The order of the shoulder positions was randomized. The arm was manually supported by the research assistant in the testing position which was confirmed with a standard goniometer. Once 2-3 s of resting (no-load) EMG activity was recorded, the support was removed and participants maintained the arm position while holding the weight for 5 s. Participants were familiarized with the testing procedures prior to data collection. Each participant was asked to keep their elbow extended but not locked. One exertion was performed in each position and a one minute break was given between exertions to minimize muscle fatigue.

**Figure 3.**
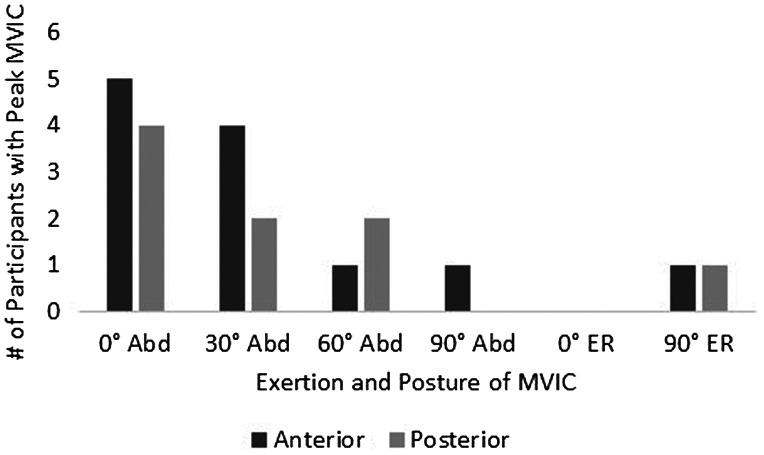
Summary of exertion and posture which demonstrated peak activity during MVIC for anterior and posterior regions. Abd = humeral abduction; ER = external rotation.

EMG signals were recorded using a NORAXON EMG system (Telemyo 2400T, Noraxon, Scottsdale, AZ, USA) at a sampling rate of 3000 Hz. The EMG signals were amplified 500X and filtered using a 10–1000 Hz band pass filter. Data were transferred to a personal computer for processing using MATLAB™ (Version R2013b 8.2.0.701). All raw EMG signals were smoothed using a root mean square (RMS) EMG envelop with a moving window of 100 ms. Each participant’s data were normalized using the peak RMS value recorded during the MVICs found for the corresponding region, regardless of which of the six positions that peak value was recorded (Figure 3). The RMS of the anterior region was divided by the RMS of the posterior to calculate the muscle activation ratio.

The ratio of the activation level for the anterior and posterior region was calculated for each participant’s arm position. The ratios were then analyzed using SPSS statistical software (version 24.0, SPSS Inc, Chicago, IL, U.S.A). Kolmogorov-Smirnov analysis confirmed that data were not normally distributed; therefore, non-parametric statistical tests were carried out. A Friedman test was conducted to evaluate differences in medians among the different arm positions. The Wilcoxon test was used for *post hoc* analysis. Bonferroni adjustments were made and statistical significance was set at *P* = 0.005. Data are reported as the median and interquartile range (IQR).

## Results

The exertions and postures which demonstrated peak activity during the MVIC for anterior and posterior regions varied between participants (Figure 3). Only two participants demonstrated peak activity during external rotation exertions, one was for the anterior region and the other for the posterior. The exertion and posture that demonstrated the highest peak RMS value most frequently was humeral abduction at 0°.

**Figure 4.**
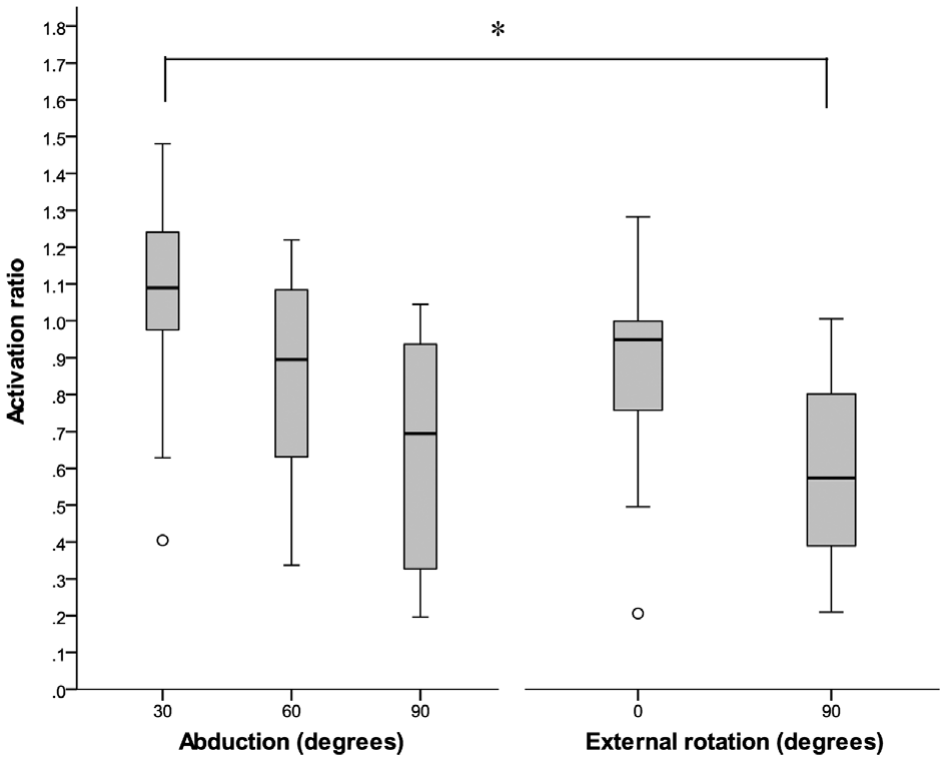
Median ratio (anterior: posterior) for abduction and external rotation exertions in various postures. Significant difference (*p* < 0.005). Outliers indicated by o.

EMG data from one participant was of poor quality for both abduction and external rotation trials. Therefore, this participant’s data were not included in the analyses. There were statistically significant differences in the median activation ratios between the different postures investigated, *χ*^2^ (4, *n* = 9) = 18.311, *p* = 0.001). The median ratio (IQR) of anterior to posterior activation level for 30°, 60°, and 90° of humeral abduction and external rotation at neutral and 90° of humeral abduction are reported in Figure 4.

Among the abduction exertions, no differences existed between 30° and 60° or 30° and 90°. However, the decrease in the median ratio between 90° abduction to 60° was close to approaching significance (*Z* = −2.666, *p* = 0.008). Among the external rotation tasks, no differences occurred between postures. Between the abduction and external rotation tasks, differences emerged between external rotation with the glenohumeral joint in 90° of abduction and abduction at 30° (*Z* = −2.934, *p* = 0.003). The median ratio between external rotation with the glenohumeral joint in 90° of abduction and abduction at 60° was close to approaching significance (*Z* = −2.599, *p* = 0.009).

## Discussion

The supraspinatus is a clinically important muscle that is frequently tested and treated for pathology. Although growing evidence suggests that the anterior and posterior regions of supraspinatus are functionally distinct (Roh et al. 2000; Kim et al. 2007, 2013; Hermenegildo et al. 2014), EMG activity of only the anterior portion of the muscle has been studied previously and this has been considered to be representative of the entire muscle. To the best of our knowledge, *in vivo* activity of the posterior region has not been assessed; thus, its contribution to modulating force transfer to the tendon is not clear. Presently, we compared the relative activity of the two regions of supraspinatus between isometric contractions in different postures that represent common shoulder testing and strengthening positions (Wilk et al. 2002; Thigpen et al. 2006; Reed et al. 2016). The findings support our hypotheses that the relative contributions of the two regions of supraspinatus differ with various isometric abduction and external rotation exertions and also that the relative contribution of the posterior region increases as the arm is elevated and externally rotated. Methodologically, the present study provides the groundwork for future EMG recordings from the two portions of supraspinatus. Additionally, our observations have important clinical ramifications on assessment and treatment approaches for rotator cuff dysfunction and highlight the need for further research exploring the regional muscle activation patterns of supraspinatus.

One of the most important contributions of the present study is the selective recording of EMG activity from the posterior region of supraspinatus. As no references exist for electrode placement in this region, we used the detailed anatomical descriptions and three-dimensional computer models of the fiber bundle architecture (Kim et al. 2007) and intramuscular innervation (Hermenegildo et al. 2014) to establish a best practice recording technique. Given the posterior region’s relatively smaller volume and its close proximity to the scapular spine, we recommend a thorough understanding of the muscle architecture be established prior to electrode insertion. Real-time ultrasound can serve as an important visual guide and ensure correct placement.

Electrode placement within the anterior region of the supraspinatus for the present study coincides with numerous previous studies (McMahon et al. 1996; Reinold et al. 2007; Boettcher et al. 2009, 2010; Allen et al. 2013) which have referenced electrode placement guidelines by Basmajian (1978), Perotto (1994), and Geiringer (1994). In the current study, we inserted the fine-wire electrodes with the needle angled (medial to lateral direction) to allow dynamic viewing of the needle tip with real-time ultrasound. Thus, our insertion point (Figure 1(B), *) may be more medial than other studies. However, the final location of the electrode tips within the anterior muscle belly agrees with previous studies.

Previous studies of the anterior region of supraspinatus have concluded it plays an important role in all phases of glenohumeral motion as a humeral rotator and as a dynamic stabilizer (Glousman et al. 1988; Wuelker et al. 1998). In the present study, we found muscle activation of both anterior and posterior regions with abduction and external rotation exertions.

Thus, the posterior region also plays a role with these exertions and in the various postures assessed. The muscle activation ratio (anterior: posterior) decreased as the participants’ humeri were elevated into higher degrees of abduction. This suggests the relative contribution of the posterior region may be particularly important in these higher elevations.

In a biomechanical study that investigated the contributions of the anterior and posterior regions to the magnitude and direction of humeral rotation in cadaveric specimens, Gates et al. (2010) concluded the distinct regions of supraspinatus had different functions with respect to humeral internal and external rotation. The anterior region was found to induce both internal and external rotation of the humerus, whereas the posterior region induced only external rotation when loaded. Although activity of the posterior region during internal rotation exertions was not investigated in our study, we found the activation ratio to be significantly lower during the external rotation exertion at 90° of abduction compared to the abduction exertions at both 60° and 90° of abduction. Our findings provide further support for those of Gates et al. (2010) and underscore the posterior region’s role in external rotation.

Clinically, strengthening exercises for the supraspinatus are an integral part of rehabilitation and conditioning programs (Dark et al. 2007; Boettcher et al. 2009). To date, these prescribed exercises have been based on EMG studies of the anterior region. Clearly, training and rehabilitation protocols that address the function(s) of the posterior region are also needed. This is especially germane to supraspinatus tendinopathy, which typically involves substantial morphological changes to the posterior region (Kim, Dahiya et al. 2010, Kim et al. 2015). Based on our findings, incorporating activities with higher degrees of humeral abduction may be important for the posterior region. When considered together with fiber-type distributions within the muscle, resistance training modes that account for the mixed fiber-type distribution may be ideal. Although both regions of the supraspinatus are composed predominantly of type I fibers (Lovering and Russ 2008; Kim et al. 2013), the percentage of type II fibers is significantly higher within the posterior region compared to the anterior (Kim et al. 2013). Nevertheless, further studies that include recordings from the posterior region are needed. For example, investigating the regional activation levels at varying speeds may assist in refining training and exercise prescription for the two regions of supraspinatus. Further, a better understanding of the timing of activation and the relationship between regional EMG activity and shoulder scapular kinematics may provide important insight into the interplay between these two distinct regions of supraspinatus.

Numerous reference exertions have been suggested to elicit maximal activation of the supraspinatus (Gowan et al. 1987; Morris et al. 1998; Boettcher et al. 2008, 2010). However, these positions were determined based on EMG electrode placement within the anterior region. To explore postures that may elicit maximal activation of the posterior region, we assessed two types of exertions: abduction and external rotation. We normalized each region’s activity to the corresponding region’s peak RMS found among the MVIC trials for each individual. There may be alternative positions not tested in this present study that could be more appropriate as a reference value to normalize the posterior region and should be further explored along with the reliability. However, until future research identifies other positions, we recommend MVIC testing for the posterior region to include abduction exertions at lower glenohumeral ranges of motion (i.e. 0° and 30°)

Our results are based on a modest sample size given the complexity of the study. To overcome this limitation, we used a more conservative *P*-value. Additionally, only supraspinatus was assessed. Thus, interpretation of the results merit caution when considering the complex interplay between all rotator cuff muscles and the overlapping tendon attachment of the posterior region of supraspinatus and infraspinatus on the middle facet of the greater tubercle.

To conclude, we conducted a novel study based on anatomical insights that describe the relative contributions of the anterior and posterior regions of supraspinatus during isometric contractions in different arm positions. Our results, support the concept of functionally distinct regions within the supraspinatus muscle; the muscle activation ratios (anterior: posterior) were dependent on the exertion type and arm posture. Our findings provide a foundation for future studies, including clinical studies comparing activity in participants with normal shoulder function to those with various shoulder pathologies, and also biomechanical modeling studies involving the supraspinatus. Further, re-evaluation of existing shoulder rehabilitation protocols is needed to reflect the divergent roles of the supraspinatus partitions. A more refined approach that addresses function of both regions may lead to improved clinical outcomes for conservative management approaches and post-surgical rehabilitation.

## Funding

This work was supported by the Physiotherapy Foundation of Canada, Alun Morgan Memorial Award in Orthopaedics.

## Disclosure statement

The authors report no financial interests or benefits.
